# Analysis of the Effect of the TRPC4/TRPC5 Blocker, ML204, in Sucrose-Induced Metabolic Imbalance

**DOI:** 10.3390/ph16081100

**Published:** 2023-08-03

**Authors:** Mizael C. Araújo, Suzany H. S. Soczek, Jaqueline P. Pontes, Bruno A. S. Pinto, Lucas M. França, Bruna da Silva Soley, Gabriela S. Santos, Warlison F. de Silva Saminez, Fernanda K. M. Fernandes, João L. do Carmo Lima, Daniele Maria-Ferreira, João F. S. Rodrigues, Nara L. M. Quintão, Valério Monteiro-Neto, Antônio M. A. Paes, Elizabeth S. Fernandes

**Affiliations:** 1 Programa de Pós-Graduação, Universidade CEUMA, São Luís 65075-120, MA, Brazil; mizaelcalacioo@outlook.com (M.C.A.); gabyiisantos9@gmail.com (G.S.S.); w.felipeloading@gmail.com (W.F.d.S.S.); fernandafernande5@hotmail.com (F.K.M.F.); joaofranciscosr@hotmail.com (J.F.S.R.); 2 Programa de Pós-Graduação em Biotecnologia Aplicada à Saúde da Criança e do Adolescente, Faculdades Pequeno Príncipe, Curitiba 80230-020, PR, Brazil; suzanyhellen@gmail.com (S.H.S.S.); daniele.ferreira@pelepequenoprincipe.org.br (D.M.-F.); 3 Instituto de Pesquisa Pelé Pequeno Príncipe, Curitiba 80250-060, PR, Brazil; 4 Programa de Pós-Graduação em Ciências da Saúde, Universidade Federal do Maranhão, São Luís 565085-080, MA, Brazil; jaquelinepessoasp@gmail.com (J.P.P.); lucas.mf@ufma.br (L.M.F.); lima98joaolucas@gmail.com (J.L.d.C.L.); valerio.monteiro@ufma.br (V.M.-N.); antonio.marcus@ufma.br (A.M.A.P.); 5 Departamento de Ciências Fisiológicas, Universidade Federal do Maranhão, São Luís 565085-080, MA, Brazil; bruno.pinto@ufma.br; 6 Departamento de Farmacologia, Universidade Federal do Paraná, Curitiba 81531-980, PR, Brazil; brunasoley@gmail.com; 7 Programa de Pós-Graduação em Ciências Farmacêuticas, Universidade do Vale do Itajai, Itajaí 88302-901, SC, Brazil; nara.quintao@univali.br

**Keywords:** high sucrose intake, metabolic changes, fat deposition, hepatic steatosis, TRPC4 and TRPC5 channels

## Abstract

Sugar-induced metabolic imbalances are a major health problem since an excessive consumption of saccharides has been linked to greater obesity rates at a global level. Sucrose, a disaccharide composed of 50% glucose and 50% fructose, is commonly used in the food industry and found in a range of fast, restaurant, and processed foods. Herein, we investigated the effects of a TRPC4/TRPC5 blocker, ML204, in the metabolic imbalances triggered by early exposure to sucrose-enriched diet in mice. TRPC4 and TRPC5 belong to the family of non-selective Ca^+2^ channels known as transient receptor potential channels. High-sucrose (HS)-fed animals with hyperglycaemia and dyslipidaemia, were accompanied by increased body mass index. mesenteric adipose tissue accumulation with larger diameter cells and hepatic steatosis in comparison to those fed normal diet. HS mice also exhibited enhanced adipose, liver, and pancreas TNFα and VEGF levels. ML204 exacerbated hyperglycaemia, dyslipidaemia, fat tissue deposition, hepatic steatosis, and adipose tissue and liver TNFα in HS-fed mice. Normal mice treated with the blocker had greater hepatic steatosis and adipose tissue cell numbers/diameter than those receiving vehicle, but showed no significant changes in tissue inflammation, glucose, and lipid levels. The results indicate that TRPC4/TRPC5 protect against the metabolic imbalances caused by HS ingestion.

## 1. Introduction

The intake of high-content sugar foods and beverages from an early age has been linked to an increased risk of obesity, type II diabetes, and cardiovascular diseases, amongst other chronic pathological alterations [1,2,3,4,5]. Sucrose is a disaccharide composed of 50% glucose and 50% fructose commonly added to foods and drinks as a sweetener [6]. In humans, the long-term daily ingestion of sucrose has been linked to an increase in body weight, fat mass, hepatic steatosis, and cholesterol levels in overweight subjects [7,8]. In addition, studies with healthy- and normal-weight young male volunteers have demonstrated that high-sucrose (HS) diets augment glucose, low-density lipoprotein, and C-reactive protein quantities [9,10]. Similar observations have been made in rodents following HS diet [11,12,13,14]. This evidence demonstrates that HS causes important metabolic imbalances, which can result in chronic pathologies such as metabolic syndrome (MS), a major health problem which affects the global population at all ages [15,16].

Transient receptor potential channels are non-selective Ca^+2^ channels involved in a plethora of pathological and physiological roles [17,18,19,20,21]. First described in *Drosophila melanogaster* (for review see: [22,23,24]), it is now known that their expressions and activation profiles on neuronal and non-neuronal cells can influence the protection against or the development of a range of chronic diseases in mammals, including pain [25,26], cardiovascular diseases [27,28], asthma [29,30], amongst others. Also, their contributions to MS have been explored within the last several decades, especially in regard to TRP vanilloid 1 (TRPV1) and TRP ankyrin 1 (TRPA1) channels [31,32]. On the other hand, information on the involvement of TRP canonical channels 4 (TRPC4) and 5 (TRPC5) in weight and metabolic regulations remains scarce and deserves further investigation. As TRPC4 and TRPC5 can form homo- and heterotetramers between themselves and also with TRPC1, their roles in metabolic homeostasis are rather complex and have been recently discussed [33]. Interestingly, TRPC4 activation was found to modulate insulin secretion by triggering pancreatic cell depolarisation and Ca^2+^ influx, and the disruption of TRPC1/TRPC5 signalling was demonstrated to result in an increased generation of adiponectin by adipocytes [34,35]. Recent studies have also demonstrated that the central actions of liraglutide and semaglutide, type II antidiabetic drugs associated with marked weight loss, on hypothalamic proopiomelanocortin (POMC) neurones require TRPC5 signalling [36,37]. On the contrary, the use of TRPC4/TRPC5 inhibitors was requested for cosmetic weight loss and treatment of obesity, type II diabetes, MS, and hepatic steatosis (accession numbers: WO/2018/146485; EP3579838; US20200345741).

Herein, in order to obtain more information on the role of TRPC4 and TRPC5 in the metabolic alterations caused by HS diet, we used a dual TRPC4/TRPC5 blocker, ML204, and investigated its effects on body weight, hyperglycaemia, dyslipidaemia, fat tissue accumulation, and tissue inflammation, in comparison with mice fed a standard diet. The results indicate a protective role for TRPC4/TRPC5 against the metabolic imbalances caused by HS ingestion.

## 2. Results

### 2.1. High Sucrose Induces Increased Body Mass Index, Fat Accumulation, and Glycaemia 

**Figure 1:**
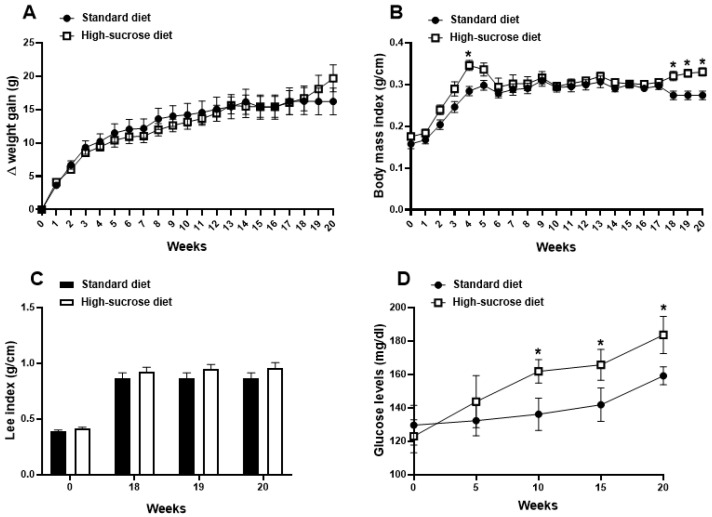
Effects of high-sucrose (HS) diet on body weight, body mass (BMI), and Lee indexes, and glycaemia. Animals received either HS or standard diet for 20 weeks (*n* = 6/group). Body weight gain (**A**) and BMI (**B**) were registered once a week. Lee index (**C**) was measured once a week from the 18th week, and blood glucose levels (**D**) were recorded at every 5 weeks. Glucose was measured in non-fasted animals. * *p* < 0.05, differs from the standard diet group.

We initially investigated the effects of HS intake on body weight, BMI, fat mass, and glycaemia, parameters that are commonly affected by diet (for review see: [38,39]). Although only a modest increase in body weight at the 20th week was observed in animals fed HS diet in comparison with those receiving standard chow (Figure 1A; *p* > 0.05), HS ingestion led to a transient increase in BMI at the 4th week, which became sustained from the 18th week in comparison with animals receiving a standard diet (Figure 1B; *p* < 0.05). An analysis of the areas under the curves from the 18th to the 20th week demonstrated that there are no differences in body weight between groups (*p* > 0.05): 32.5 ± 9.8 (standard diet group) versus 34.7 ± 9.6 (HS diet group). In addition, an analysis of the areas under the curves from the week 18th to the 20th week confirmed the differences observed in Figure 1B between the BMIs of HS- and standard diet-fed mice as follows: 0.549 ± 0.02 (standard diet group) versus 0.653 ± 0.019 (HS diet group); *p* < 0.05. Hyperglycaemia was also noted from the 10th week in HS-fed mice (Figure 1D; *p* < 0.05) when compared to those receiving standard diet. On the other hand, Lee indexes were similar in those receiving either diets (Figure 1C; *p* > 0.05). The mesenteric fat was collected and weighed at the end of the 20th week as a measure of adipose tissue accumulation in the abdomen. HS diet-fed animals with a marked fat deposit increase in comparison to those receiving standard diet showed the following mean ± SEM values: 10.8 + 1.9 (standard diet group) versus 24.3 ± 1.8 mg of fat/g of body weight (HS diet group; *p* < 0.05).

### 2.2. ML204 Regulates Circulating Glucose and Lipid Levels

**Figure 2:**
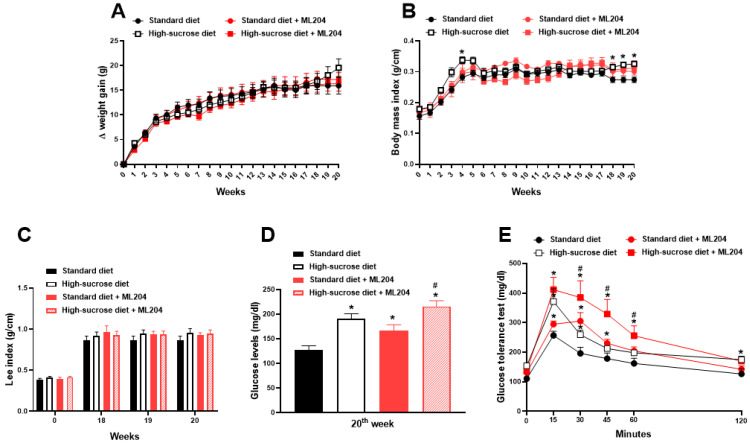
Repeated ML204 treatment increases blood glucose levels and impairs glucose tolerance. Animals received either a high-sucrose or standard diet for 20 weeks (*n* = 8/group). Body weight gain (**A**) and body mass index (BMI; (**B**)) were registered once a week. Lee index (**C**) was measured once a week from the 18th week. Blood glucose levels (**D**) were recorded at every 5 weeks and a glucose tolerance test (**E**) was performed at the 20th week. Glucose and glucose tolerance were measured in non-fasted and fasted animals, respectively. ML204 (2 mg/kg) or vehicle (3% dimethyl sulfoxide (DMSO) in phosphate-buffered saline (PBS)) was administered subcutaneously, once a day, for 7 days starting at the beginning of the 19th week. * *p* < 0.05, differs from the standard diet group; ^#^ *p* < 0.05, differs from the high-sucrose diet control group.

In order to assess the involvement of TRPC4/TRPC5 in the metabolic changes caused by a high sugar diet, animals receiving a standard or HS diet were treated with either ML204 or vehicle. Figure 2 depicts the effects of ML204 on HS- and standard diet-fed mice. Repeated treatment with ML204 did not affect body weight gain, nor body mass (BMI) and Lee indexes in animals fed any of the diets (Figure 2A–C; *p* > 0.05). In contrast, ML204 further increased hyperglycaemia and impaired glucose tolerance in HS-fed mice (Figure 2D,E; *p* < 0.05). Of notice, ML204 also induced an elevation of blood glucose and caused glucose intolerance in those receiving a standard diet (Figure 2D,E; *p* < 0.05).

**Figure 3:**
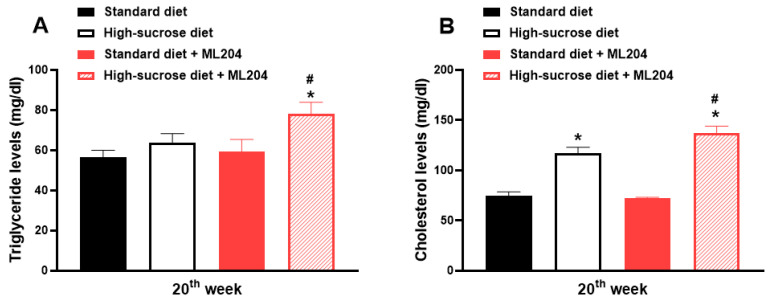
Repeated ML204 treatment causes hypertriglyceridaemia and exacerbates hypercholesterolemia in animals fed a high-sucrose (HS) diet. Animals received either HS or standard diet for 20 weeks (*n* = 8/group). Triglyceride (**A**) and total cholesterol (**B**) levels were measured at the end of the 20th week. ML204 (2 mg/kg) or vehicle (3% DMSO in PBS) was administered subcutaneously, once a day, for 7 days starting at the beginning of the 19th week. * *p* < 0.05, differs from the standard diet group; ^#^ *p* < 0.05, differs from the high-sucrose diet control group.

Interestingly, ML204 did not alter triglyceride or cholesterol levels and neither the amount of mesenteric fat in standard diet-fed mice (Figure 3A,B; *p* > 0.05). Conversely, it caused hypertriglyceridaemia and exacerbated hypercholesterolemia in animals fed HS diet (Figure 3A,B; *p* < 0.05).

### 2.3. ML204 Increases High-Sucrose-Induced Adipose Tissue and Liver Inflammation by Modulating TNFα Production

**Figure 4:**
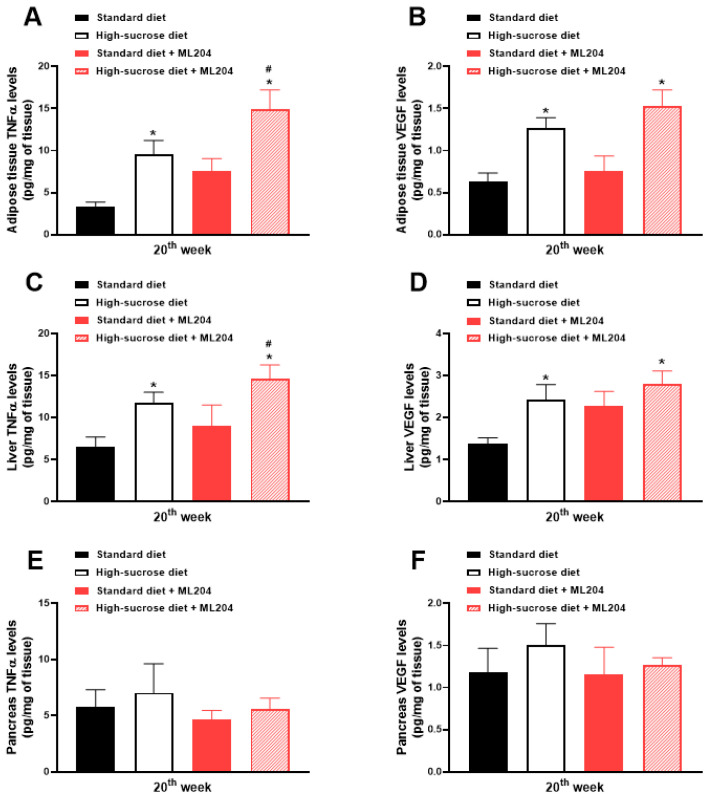
Repeated ML204 treatment enhances inflammation in the adipose and liver tissues of animals fed a high-sucrose (HS) diet. Animals received either HS or standard diet for 20 weeks (*n* = 8/group). TNFα was measured in (**A**) adipose tissue, (**C**) liver, and (**E**) pancreas. Adipose (**B**), liver (**D**), and pancreas (**F**) VEGF tissue levels. Samples were collected at the end of the 20th week. ML204 (2 mg/kg) or vehicle (3% DMSO in PBS) was administered subcutaneously, once a day, for 7 days starting at the beginning of the 19th week. * *p* < 0.05, differs from the standard diet group; ^#^ *p* < 0.05, differs from the high-sucrose diet control group.

Inflammation is an important event that contributes to metabolic imbalances; thus, tumour necrosis factor α (TNFα) and vascular endothelial growth factor (VEGF) levels (inflammatory mediators involved in obesity and glycaemia control (for review see: [33,40])) were evaluated in adipose tissue, liver, and pancreas samples obtained from animals fed either a standard or HS diet. HS diet-fed mice led to a greater release of these inflammatory mediators in adipose tissue and liver samples in comparison with those receiving standard diet, an effect which was further enhanced by ML204 injection (Figure 4A–D; *p* < 0.05). No differences were observed in regard to the pancreas levels of TNFα and VEGF between diets (standard diet versus HS diet) or treatments (vehicle versus ML204) (Figure 4E,F); *p* > 0.05.

### 2.4. ML204 Enhances Adipocyte Area and Mesenteric Fat Accumulation in High-Sucrose-Fed Mice and the Size of Adipocytes in Those Receiving Standard Diet

**Figure 5:**
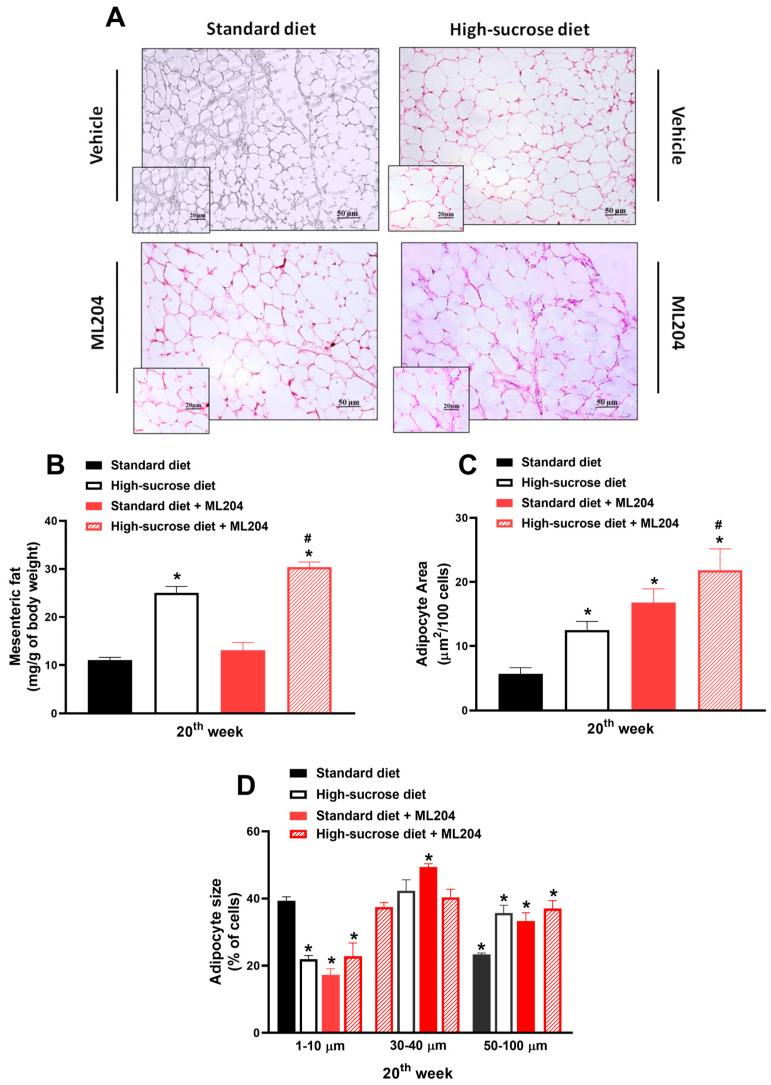
Repeated ML204 treatment causes fat accumulation in animals fed high-sucrose (HS) diet and the size of adipocytes in those receiving standard diet. (**A**) Representative H&E histology sections of adipose tissue (20 and 50 µm areas) from animals fed either HS or standard diet for 20 weeks (*n* = 8/group). Mesenteric fat/body weight ratios (**B**), adipocyte area (**C**), and size (**D**) measured at the end of the 20th week. ML204 (2 mg/kg) or vehicle (3% DMSO in PBS) was administered subcutaneously, once a day, for 7 days starting at the beginning of the 19th week. Samples were stained by haematoxylin and eosin (H&E). * *p* < 0.05, differs from the standard diet group; ^#^ *p* < 0.05, differs from the high-sucrose diet control group.

The analysis of the mesenteric adipose tissue (Figure 5A–D; *p* < 0.05) indicates a greater fat accumulation in animals fed HS diet. The mice receiving HS diet presented with higher fat-to-body weight ratios, as well as with larger adipocyte areas and sizes (>50–100 µm) than those that received standard diet; *p* < 0.05. ML204 administration further enhanced fat weight and adipocyte area in HS-fed mice without affecting the size of their adipocytes (Figure 5B–D; *p* < 0.05). Interestingly, although no effects were observed for ML204 in regard to the fat weight and adipocyte areas of mice fed standard diet, the same animals presented a significant percentage of medium-sized cells (30–40 µm; *p* < 0.05) in comparison with vehicle controls.

### 2.5. Non-Alcoholic Fatty Liver Disease Activity Score Is Enhanced by ML204 in High-Sucrose-Fed Mice

**Figure 6:**
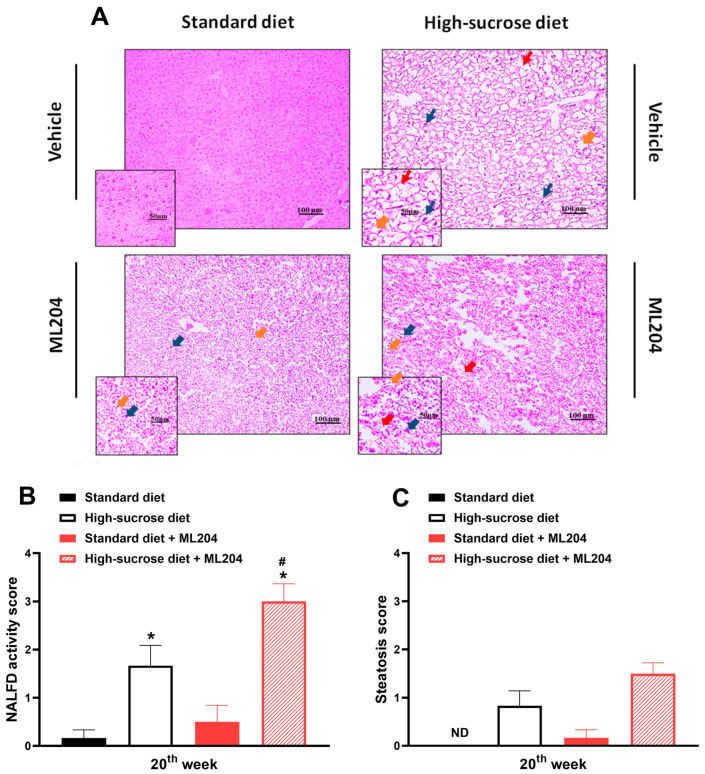
Repeated ML204 treatment increases non-alcoholic fatty liver disease (NALFD) activity score (NAS) in high-sucrose (HS)-fed mice. (**A**) Representative H&E histology sections of liver (50 and 100 µm areas) from animals fed either HS or standard diet for 20 weeks (*n* = 8/group). NAS (**B**) and steatosis score (**C**) were measured at the end of the 20th week. ML204 (2 mg/kg) or vehicle (3% DMSO in PBS) was administered subcutaneously, once a day, for 7 days starting at the beginning of the 19th week. NAS was determined by the summation of hepatic steatosis (red arrows), ballooning (orange arrows), and inflammatory cell influx (dark green arrows) scores. Samples were stained by haematoxylin and eosin (H&E). * *p* < 0.05, differs from the standard diet group; ^#^ *p* < 0.05, differs from the high-sucrose diet control group.

We next investigated the effects of ML204 on non-alcoholic fatty liver disease NAFLD activity score (NAS). Figure 6A–C show that HS diet increases NAS by causing hepatic steatosis (red arrows), ballooning (orange arrows), and inflammatory cell influx (dark green arrows) to the tissue; *p* < 0.05. In a much lesser degree, ballooning and cell migration, but not steatosis are noted in standard-diet animals treated with ML204 (Figure 6; *p* < 0.05).

## 3. Discussion

In the last several decades, foods and beverages containing high sugar levels became popular products found in the shelves of houses, restaurants, bars, and supermarkets all around the world. Diets rich in such products are now well-known to increase the risk for chronic diseases associated with metabolic imbalances, including obesity [38,41,42]), type II diabetes [42], and cardiovascular diseases [43], amongst other chronic pathological alterations, indicating that HS diets cause important metabolic changes, which can result in MS. This is particularly important considering the early age intake of HS [1,2,3,4,5]. Indeed, a recent analysis of the global scenario demonstrated that nearly 3% of children and 5% of the adolescent population present with MS [16]. Also, a drastic panorama has been noted in the adult population, with a 19.5% and 48.6% prevalence of MS amongst 20–39 year olds and those ≥60 years, respectively [15].

Herein, as expected [44,45,46,47,48], HS diet promoted a modest weight gain. Also in agreement, HS significantly increased BMI, hyperglycaemia, mesenteric fat deposition, glucose tolerance, hypercholesterolemia, and NAS score in comparison with mice receiving a standard diet. In addition, HS-fed mice presented with adipose tissue and liver inflammation characterised by increased levels of TNFα, a cytokine involved in adipocyte hypertrophy, fat deposition, insulin resistance, and hyperglycaemia (for review see: [33]). Higher VEGF levels were also noted in both liver and adipose tissue samples from HS mice. Of note, VEGF production in the adipose tissue is suggested to play a protective role against hypoxia, insulin resistance, and obesity, being an essential growth factor in the maintenance of metabolism homeostasis [49,50]. 

We show for the first time that when repeatedly administered to mice, ML204 exacerbates hyperglycaemia, dyslipidaemia, fat tissue deposition, hepatic steatosis, and adipose tissue and liver inflammation in HS-fed mice. The compound also affected normal mice, promoting hepatic steatosis and increasing adipose tissue cell numbers and diameter without causing tissue inflammation, or changing glucose and lipid levels.

We used a dual TRPC4/TRPC5 blocker, ML204, to further understand the roles of TRPC4 and TRPC5 in the metabolic alterations caused by HS diet. ML204 was first described as a potent and selective TRPC4/TRPC5 inhibitor in vitro [51,52]. Since then, accumulating evidence has demonstrated its ability to modulate inflammatory responses by regulating cytokine (TNFα, IL-1β, IL-6, and IL-10) production in vivo and in vitro, although reductions in or upregulations of these proteins seem to depend on the model used [53,54,55]. Importantly, inflammation, alongside oxidative stress (events in which these channels are shown to modulate) in metabolic tissues, are important mechanisms of glucose and lipid imbalances, which can ultimately contribute to MS development and progression (for review see: [33]).

Knowledge from the last several decades has consistently pointed to the importance of TRP channels such as TRPV1 and TRPA1 in MS [31,32]. However, only recently, an emerging and complex role has been attributed to TRPC channels, especially TRPC5. This is mainly due to its ability to form not only homotetrameric, but also heterotetrameric complexes with other TRP channels (TRPC4 and TRPC1; for review see: [33]).

In this context, it is important to consider the contributions of both homo- and heterotetramers containing TRPC5 in the regulation of metabolism. Whilst TRPC4 activation was shown to modulate insulin secretion, and TRPC1/TRPC5 signalling was suggested to regulate adiponectin release by adipocytes [34,35], TRPC4/TRPC5 inhibitors were recently patented for use in weight loss, type II diabetes, MS, and hepatic steatosis (accession numbers: WO/2018/146485; EP3579838; US20200345741). Also, TRPC5 was suggested as a key mediator for the central effects of the glucagon-like peptide-1 (GLP-1) receptor agonists liraglutide and semaglutide [34,35], used for type II diabetes and weight loss [56,57]. The above pieces of evidence indicate that TRPC5 contributions to metabolic homeostasis may require both central and peripheral actions, and depend on the tetramer activation. Nonetheless, the results clearly indicate that TRPC4/TRPC5 channels protect against the metabolic imbalances caused by HS ingestion.

## 4. Materials and Methods

### 4.1. Animals

Inbred male and female C57BL/6 mice (3 weeks old) from the Biological Service Unit of Universidade CEUMA were used. All the animals were housed under 12 h light/dark cycle, at a controlled environmental temperature (21 ± 2 °C) and humidity (60 ± 5%). All the experimental groups were matched for gender and body weight. All experiments followed the recommendations of the Brazilian guidelines on animal experimentation of the National Council for the Control of Animal Experimentation (CONCEA) and the ARRIVE guidelines [58]. All procedures were previously approved by the Animal Use Ethics Committee of Universidade CEUMA (protocol n^o^ 00081/18).

### 4.2. High-Sucrose-Induced Metabolic Disturbances

A total of 22 female and 22 male mice were used in the study. In order to standardise the model, mice received either normal or HS diet (3 male and 3 female mice/group), as previously described [59]. Either standard (Nuvital^®^, Nuvilab; Curitiba; Brazil; 3.52 kcal/g; composed of 55.4% carbohydrate (10% sucrose), 21% protein, and 5.2% lipids) or HS diet (3.48 kcal/g; composed of 65% carbohydrate (25% sucrose), 12.3% protein, and 4.3% lipids) was fed to the mice for 20 weeks. Both water and food were provided ad libitum. Body weights and body mass indexes (BMI; body weight (g)/ nose-to-anus length (cm)) were registered for each mouse prior to and at every week, once a week post-diet. Analyses of AUCs were performed for body weight changes and BMIs between weeks 18 and 20. In parallel, the Lee index (∛body weight (g)/nose-to-anus length (cm) × 1000) [59]) was analysed at the 18th, 19th, and 20th weeks post-diet. Glycaemia was measured at baseline and at every five weeks from blood samples collected from the tail veils of restrained non-fasted animals by using a portable glucose meter and glucose strips (Accu-Chek Active^®^, Roche, Indianapolis, IN, USA).

To evaluate the effects of ML204, standard- and HS diet-fed mice received either vehicle (3% DMSO in PBS; *v*/*v*) or ML204 (2 mg/kg), subcutaneously, once a day, for 7 days starting at the beginning of the 19th week. Body weights, body mass, and Lee indexes were measured at baseline, prior to and after ML204 treatment (between the 18th and 20th weeks), and evaluated as described above. Also, at the end of the 20th week, following 8 h of fasting, a glucose tolerance test was performed. Just before fasting, blood glucose levels were tested. Then, the animals were anaesthetised with ketamine and xylazine (75 mg/kg and 1 mg/kg, respectively). Blood samples were collected by cardiac puncture for analysis of the circulating quantities of triglyceride and cholesterol. Liver, mesenteric adipose, and pancreas tissue samples were collected for histology and inflammatory mediator production analysis (TNFα and VEGF).

### 4.3. Glucose Tolerance Test

For analysis of glucose tolerance, baseline blood glucose was measured in fasted mice. Then, animals received an intraperitoneal injection of glucose (2 g/kg; [60]). Next, glycaemia was measured from blood samples collected from the tail veil of restrained mice at 15, 30, 45, 60, and 120 min post-glucose administration in comparison to baseline by using a portable glucose meter and glucose strips (Accu-Chek Active^®^, Roche, Indianapolis, IN, USA).

### 4.4. Cholesterol and Triglyceride Quantifications

The blood collected at the end of the 20th week post-diet was centrifuged at 1300× *g* for 15 min, for serum separation. Serum levels of cholesterol and triglyceride were measured by using commercial kits according to manufacturer’s instructions (Labtest, MG, Brazil). For this, 10 µL of the serum was used per reaction for each assay in duplicate. Samples were incubated at 37 °C for 10 min and then, the absorbances were read at 500 nm. The results are expressed as milligram per decilitre (mg/dL) of cholesterol or triglyceride.

### 4.5. TNFα and VEGF Tissue Levels

Approximately 100 mg of tissue was homogenised in 500 µL of PBS containing protease inhibitors (cOmplete™, EDTA-free Protease Inhibitor Cocktail; Sigma-Aldrich; São Paulo; Brazil), by using a tissue lyser (6 cycles of 30 s each, 4000 r.p.m.; between cycles, samples were kept on ice for 20 s; TissueLyser LT; Qiagen; São Paulo, SP, Brazil). The homogenates were centrifuged at 1000 r.p.m., for 10 min, at 4 °C and the supernatants collected and used for the measurements of TNFα and VEGF in adipose tissue, liver, and pancreas samples by using pre-coated plates, as per the manufacturer´s protocol (Sigma-Aldrich; São Paulo, SP, Brazil). Protein content of each supernatant was determined by using BCA protein kit, according to manufacturer´s instructions (Sigma-Aldrich; São Paulo, SP, Brazil). For this, 100 µL per well of the supernatants was used for each assay in duplicate. Absorbances for each sample were compared to those of a standard curve of each inflammatory mediator. The results are expressed as picograms of sample per milligram (pg/mg) of tissue.

### 4.6. Histology

Portions of the liver and mesenteric adipose tissues were collected, washed in PBS, and infused with 10% formalin in PBS for 24 h. Then, the samples were embedded in paraffin for cutting. Tissue samples were deparaffinised in xylene followed by dehydration in a graded series of ethanol/water. Serial 10 µm (adipose tissue) and 5 µm (liver) sagittal sections were cut on a microtome. Samples were stained with haematoxylin and eosin (H&E) to allow for the observation of the general morphology of tissues by microscopy (Nikon Eclipse Ci-L; Nikon, Biolab; São Paulo; Brazil).

Two independent observers blinded to treatments scored the liver sections for steatosis (score 0–3), hepatocyte ballooning (score 0–2), and inflammation (score 0–3), according to NAS, modified [14]. The maximum possible score was 8, and the results are expressed as the mean of the scores attributed by each observer.

Four pictures from separate parts of each section of adipose tissue were taken. Then, the area of 100 cells was measured (ImageJ; bundled with Zulu OpenJDK 13.0.6.; Madison, WI, USA) and the percentage (%) of the different cell sizes was calculated [45]. All counts were performed by two different observers blinded to treatments.

### 4.7. Statistical Analysis

The results are presented as mean ± mean standard error (SEM). For multiple statistical comparisons between groups, data were analysed by repeated measures analysis of variance (ANOVA), or one-way ANOVA, followed by the Bonferroni test with FDR correction. Unpaired t tests were used when appropriate. Histology scores were analysed using Kruskal–Wallis test followed by Dunn’s test for multiple comparisons. All data were analysed in GraphPad Prism 6.0. (now Dotmatics; Woburn, MA, USA); *p* < 0.05 was considered significant. All n numbers are indicated on the graphs.

## 5. Conclusions

Although we were not able to dissect the expression sites in which activated TRPC4 and TRPC5 can influence metabolism changes, the data presented herein clearly demonstrated the importance of TRPC4/TRPC5 as protective channels against the metabolic imbalances caused by HS diet and highlights the need for further investigations in the field. Considering the complex roles of TRPC4 and TRPC5 either as homotetramers or heterotetramers, as well as of other heterotetramers formed between TRPC5 and TRPC1, for example, it is essential to highlight that the interpretation of data obtained from the use of TRPC5 activators, blockers, or genetically modified animals must always take into account such receptor interactions.

## Data Availability

Data is contained within the article.
